# Multilocus Sequence Typing Unveils Two Novel Genospecies of *Borrelia burgdorferi* Sensu Lato in Ticks Infesting Cricetid Rodents of Northern Chile

**DOI:** 10.1155/tbed/8841276

**Published:** 2025-07-10

**Authors:** Catalina Parragué-Migone, Adriana Santodomingo, Richard Thomas, Sebastián Muñoz-Leal, Gerardo Acosta-Jamett

**Affiliations:** ^1^Departamento de Ciencia Animal, Universidad de Concepción, Chillán, Chile; ^2^Centro de Investigación de Estudios Avanzados del Maule (CIEAM), Vicerrectoría de Investigación y Postgrado, Universidad Católica del Maule, Talca, Chile; ^3^Instituto de Medicina Preventiva Veterinaria and Center for Disease Surveillance and Evolution of Infectious Diseases, Universidad Austral de Chile, Valdivia, Chile

**Keywords:** arid-ecosystems, genetic typing, *Ixodes*, spirochetes, vector-borne microorganisms

## Abstract

Tick-borne spirochetes of the genus *Borrelia* are maintained in enzootic transmission cycles involving wild vertebrates such as rodents. The genus includes the lyme disease group (LDG), transmitted by hard ticks (Ixodidae), and the relapsing fever group (RFG), mostly transmitted by soft ticks (Argasidae). While research on *Borrelia* spirochetes has been largely concentrated in the Northern Hemisphere, recent studies have uncovered new genospecies in South American ecosystems. Particularly in Chile, while *Borrelia chilensis* is the sole species that has been cultured, multiple under characterized strains have been detected in wild rodents and ticks. This study aimed to genetically characterize strains of *Borrelia* in ticks parasitizing *Phyllotis darwini*, an abundant rodent species inhabiting the central north of the country. From 2021 to 2023, rodents were captured at two sites in the Coquimbo Region. Observed ticks were collected, morphologically identified, and submitted to DNA extraction to further detect the presence of *Borrelia* spirochetes through nested PCR targeting the *flaB* gene. Multilocus sequence typing (MLST) of eight housekeeping genes was subsequently performed on positive samples. Pairwise nucleotide comparisons and phylogenetic analyses with the retrieved sequences were conducted using maximum likelihood (ML) and Bayesian inference (BI) methods. A total of 634 *P. darwini* were captured, yielding 134 ticks, all identified as *Ixodes* spp. Ten ticks genetically identified as *Ixodes abrocomae* or *Ixodes sigelos* tested positive for *Borrelia* spp. Genetic identity and phylogenetic analyses revealed the presence of two novel LDG genospecies in Chile, where *B. chilensis* was the sole previously known species of the group. Although the vectors and pathogenic roles of these novel genospecies are currently unknown, our study underscores the need for further isolation attempts of the strains to assess their impact on wildlife or human health.

## 1. Introduction

The genus *Borrelia* comprises spirochetes that thrive in enzootic transmission cycles involving wild vertebrates and bloodsucking arthropods, such as soft ticks (Argasidae) and hard ticks (Ixodidae) [1, 2]. Ticks acquire the spirochetes after feeding on infected vertebrates [3]. Depending on the species, *Borrelia* can infect the tick progeny through transovarial transmission [3]. However, in some cases, spirochetes are not transmitted transovarially; therefore, the perpetuation in subsequent tick generations depends on chronically infected vertebrate hosts, which serve as reservoirs and sources of infection [3]. *Borrelia* spirochetes have been traditionally separated into two major groups according to the diseases they cause in humans. The lyme disease group (LDG), or *Borrelia burgdorferi* sensu lato, is transmitted by hard ticks (Ixodidae) of the genus *Ixodes*, and the relapsing fever group (RFG) transmitted mostly by soft ticks (Argasidae) of the genus *Ornithodoros* [3]. While rodents constitute vertebrate reservoirs of the spirochetes in nature, the discovery of *Borrelia* spp. in ticks associated with reptiles, birds, echidnas, and bats has brought to light a broader range of animals involved in the transmission cycles [4–6]. While the diversity of these bacteria grows, its taxonomic status is still controversial, since the genus *Borrelliella* has been proposed to classify species of the LDG [7, 8]. Because the debate regarding this classification is still open, here we opted for a conservative criterion and considered all the spirochetes transmitted by ticks as *Borrelia*.

Cultivating *Borrelia* spp. is a challenging task and requires specialized laboratories [9]. Therefore, molecular biology techniques aiming to detect and genetically identify the spirochetes in vertebrates or vectors through DNA sequencing are widely performed [10]. In fact, multilocus sequence typing (MLST) designed for eight housekeeping genes has allowed to infer evolutionary relationships between widely distributed pathogenic strains, or describe new genospecies [11, 12]. In ecosystems where the circulation of *Borrelia* spp. is unknown, the identification of these bacteria in ticks should be a primary task in order to recognize natural foci and implement further isolation attempts.

Most of the studies on LDG and RFG *Borrelia* have been performed in the Northern Hemisphere [12, 13]. However, in recent years, interest in *Borrelia* spp. has reemerges in South America. Indeed, last decade investigations demonstrated a growing number of genospecies with unknown pathogenic roles [14–19]. Particularly in Chile, the finding of *Borrelia* spirochetes is recent. First attempts to detect the bacteria were performed 25 years ago, but with negative results [20]. In 2014, *Borrelia chilensis* was isolated from *Ixodes stilesi*, a tick whose immature stages feed on rodents, while adults parasitize wild deer in the southern temperate forests of the country [21]. Noteworthy, to date it is the sole species that has been successfully cultured [14]. Furthermore, experimental transmission studies have also identified an RFG genospecies named “*Candidatus* Borrelia octodonta” in allusion to its tick vector *Ornithodoros octodontus*, a parasite of rodents in arid landscapes [17, 22]. Meanwhile, several *Borrelia* strains that circulate in wild rodents and their ticks along the country have partial genetic characterization and require a formal identification [23, 24]. The aim of this study was to identify two of those genospecies based on an MLST scheme.

## 2. Materials and Methods

### 2.1. Site of Study, Capture of Vertebrates, and Collection of Ticks

**Figure 1:**
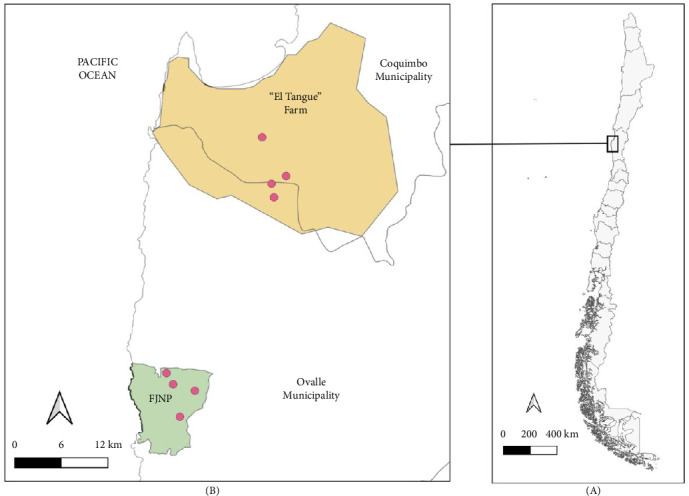
Map of the study area in the Coquimbo region in North Central Chile (A), indicating the two areas, where *P. darwini* were sampled. In each area (i.e., El Tangue Farm, in orange; Fray Jorge National Park-FJNP, in green), four trapping grids (red dots) were established (B).

The samples analyzed in this study derive from a research project that used *Phyllotis darwini* (Cricetidae) as a model species to assess the impact of environmental anthropization on its physiology, immunology, and pathogen infection. Consequently, although more species of rodents were captured, only *P. darwini* were considered. The study was performed in two sites of the Coquimbo Region, from spring of 2021 to autumn of 2023, as follows: (i) a natural area in Fray Jorge National Park (−30.642157°, −71.654061°; elevation 217 m) and (ii) a rural area “Tangue” (−30.357992°, −71.527046°; 130 m) (Figure 1). Rodents were captured with 200 Sherman traps per night, baited with oat and vanilla at each site. The traps were set at sunset (19:00–21:00 h) and checked the following morning (6:00–8:00 h). The animals were removed from the traps in plastic bags, weighed, and anesthetized with an intramuscular injection of ketamine (44 mg/kg) and xylazine (6 mg/kg). Sedated animals were inspected for ticks, which were collected with tweezers and preserved in 1.5 mL tubes in absolute ethanol. Rodents were marked with ear tags to recognize recaptures and released in the same capture site once recovered from sedation. Recaptures were excluded from the records. While nymphs and female ticks were morphologically identified using taxonomic keys [21], larvae were excluded from the analyses and deposited in the “Colección Chilena de Garrapatas” (CCG) at the University of Concepción, Chillán, Chile. Licenses for capture and procedures with animals are stated in the “Ethical Approval” section.

### 2.2. DNA Extraction, Gene Amplification, and Sequencing

Nymphs and female ticks were submitted to genomic DNA extraction individually with the Omega E.Z.N.A. Tissue DNA kit (Omega Bio Tek, Norcross, USA). DNA was quantified and assessed for quality using an Epoch Microplate Spectrophotometer (BioTek Instruments, Inc., Winooski, VT, USA). Samples with an A260/A280 DNA ratio ranging from 1.6 to 2.2 were considered suitable for downstream analyses [25]. A conventional PCR assay targeting a fragment of the tick mitochondrial 16S rRNA gene [26], was initially performed to check for inhibitors. After that, a nested PCR protocol targeting the *Borrelia flaB* gene was implemented as stated elsewhere [27].

PCR assays were performed in a final volume of 25 μL composed of 12.5 μL of DreamTaq Green PCR Master Mix (Thermo Scientific, USA), 1 μL of each primer (10 pmol), 15–50 ng of DNA, and ultrapure water. Nested rounds were implemented using 1 μL of the first-round products. *Borrelia anserina* PL [28] and DNase-free water were used as positive and negative controls, respectively. Amplicons were verified in 1.5% agarose gels stained with RedGel (Biotium Inc., Tehran, Iran), and visualized through UV light. All amplicons of expected size generated in this study were purified and Sanger-sequenced in both directions at AUSTRALomics (Valdivia, Chile) with the same primers of the PCR reactions. AB1 files were visualized, quality-checked, and primer-trimmed using Geneious Prime version 2021.2.2 (www.geneious.com). Base calls with Phred scores ≥20 were deemed suitable for further analysis [29].

Sequences of mitochondrial 16S rDNA of *Borrelia*-positive ticks, and the *Borrelia flaB* gene, were submitted to BLASTn analyses [30] to genetically identify the ticks and classify *flaB* haplotypes into the LDG or RFG. An MLST targeting eight housekeeping genes (*clpA*, *clpX*, *pepX*, *pyrG*, *recG*, *nifS*, *rplB*, and *uvrA*) was performed using primers stated in Margos et al. [10] for the LDG and primers from the *Borrelia* pubMLST database (http://pubmlst.org/borrelia) in the case of RFG. PCR master mix, sequencing procedures, and analysis of MLST sequences were performed as stated above. Sequences generated in this study were deposited in GenBank (https://www.ncbi.nlm.nih.gov/genbank/) and in the *Borrelia* pubMLST database (https://pubmlst.org/organisms/borrelia-spp).

### 2.3. Genetic Identities and Phylogenies

To evaluate if the spirochetes detected in this study belonged to different genospecies or strains of one genospecies, intraspecific genetic divergence analyses of *Borrelia* spp. were performed using available MLST datasets. For that purpose, alignments of concatenated MLST genes for 3532 strains were downloaded from the *Borrelia* PubMLST database (https://pubmlst.org/bigsdb?db=pubmlst_borrelia_isolates) on February 7, 2025. The analysis included strains of the following species: *Borrelia afzelii*, *Borrelia bavariensis*, *Borrelia bissettiae*, *B. burgdorferi* sensu stricto, *Borrelia carolinensis*, *Borrelia garinii*, *Borrelia lusitaniae*, *Borrelia valaisiana*, and *Borrelia yangtzensis*. Strains with mixed species alleles were confirmed by checking the records of the PubMLST database and were excluded from downstream analyses.

Consensus sequences of tick mitochondrial 16S rRNA and *Borrelia flaB* genes obtained in this study were compared with orthologous sequences with BLASTn. An alignment of a subset of orthologous sequences of *flaB* downloaded from GenBank and the sequences generated in this study was constructed with MAFFT [31]. A second alignment with concatenated MLST genes of LDG and RFG *Borrelia* spp. downloaded from the *Borrelia* PubMLST database was implemented in that same software. MLST gene sequences of *B. chilensis* IS9, “*Candidatus* Borrelia caatinga” PCST, “*Candidatus* Borrelia mimona” CaBmimona, and “*Ca*. B. octodonta” GP1, were downloaded from GenBank. Both alignments were curated with block mapping and gathering with entropy (BMGE) to identify informative regions for phylogenetic analyses [32].

Phylogenetic trees were constructed with maximum likelihood (ML) and Bayesian inference (BI) in IQ-TREE v. 1.6.12 [33] and MrBayes v. 3.2.6 [34], respectively. Datasets were partitioned by codon position (position 1, position 2, and position 3) [35]. The best-fit evolutionary model and partitioning scheme for ML analysis were calculated by implementing the command “-m TESTNEWONLYMERGE -mrate G” in ModelFinder [36]. Rapid hill-climbing and stochastic disturbance methods with 1000 ultrafast bootstrapping pseudoreplicates were applied to assess the robustness of the tree. Ultrafast bootstrap values of <70%, 70%–94%, and ≥95% were interpreted as nonsignificant, moderate, and strong statistical support, respectively [37]. For BI, the best partition schemes were calculated with the ModelFinder and MrBayes command “lset = mixed rates = invgamma” [38]. Two separate tests of 20,000,000 generations and four Markov chain Monte Carlo (MCMC) chains were conducted, sampling every cycle until convergence, which was assessed using Tracer v. 1.7.1 [38]. Nodes with Bayesian posterior probabilities (BPPs) >0.70 were considered of high statistical support [39]. All best-fit models and partition schemes were selected according to the Bayesian Information Criteria (BIC). Trees were visualized in FigTree v. 1.4.1 (http://tree.bio.ed.ac.uk/software/figtree/) and edited with Inkscape v. 1.1 (https://inkscape.org/). Consensus trees for both ML and BI were calculated as stated in Santodomingo et al. [17].

## 3. Results

### 3.1. Captured Rodents and Collected Ticks

Overall, 634 *P. darwini* were captured. One hundred and thirty-four ticks (44 larvae, 72 nymphs, and 18 females) were collected, with 8.99% (57/634) of the rodents parasitized (Supporting Information 1: Table S1). Although all ticks were morphologically identified as *Ixodes* spp., species-level identification was not possible to achieve because anatomical structures of taxonomic value, such as the hypostome, basis capitulum, or coxae were damaged. All larval specimens were deposited in the CCG with allotment numbers CCG 88–122.

DNA extractions from nymphs and female ticks yielded high-quality DNA (data not shown). Amplicons of the expected size were obtained after tick mitochondrial 16S rRNA gene PCR in all samples, confirming the success of the DNA extractions, and the absence of PCR inhibitors. Ten samples (seven nymphs and three females) tested positive for *flaB* nested PCR, and Sanger sequencing of the amplicons unveiled haplotypes of the LDG and RFG groups. Success in MLST gene amplification was variable, since four to eight genes were sequenced in eight samples. Mitochondrial 16S rDNA amplicons of *Borrelia*-positive ticks were sequenced in nine samples (Table 1). Sample B55 yielded tick mitochondrial 16S rDNA sequences of low quality and was discarded from further analyses.

### 3.2. Genetic Identities and Phylogenetic Trees

BLASTn analyses of mitochondrial 16S rDNA sequences of positive ticks matched *Ixodes abrocomae* and *Ixodes sigelos* with identities ranging from 99.26% to 100%. Sequences of *flaB* belonging to the LDG showed the highest similarity of 98.04%–100% with orthologous sequences retrieved previously from *Ixodes* ticks and cricetid (Cricetidae) rodents in Chile (Supporting Information 1: Table S2). The closest match with a validly described species was *B. chilensis*, with 96.41% and 96.73% similarity. The *flaB* haplotype from the RFG was identical to “Ca. B. octodonta” (Supporting Information 1: Table S2). The minimal intraspecific genetic identity calculated for the eight concatenated housekeeping genes of strains retrieved from the *Borrelia* PubMLST database was 97.66% (Table 2). This value served as a threshold to determine whether the strains identified in this study belonged to the same or different genospecies. Four samples (B45, B72, B77, and B89) had complete MLST sequences and could be fully compared against this threshold. Nucleotide pairwise comparisons of a concatenated alignment of these sequences supported the presence of two genospecies. Indeed, three samples (B45, B72, and B89) showed a minimal genetic identity of 98.76% between them, indicating they belonged to the same genospecies. In contrast, sample B77 exhibited 95.98%–96.28% identity with samples B45, B72, and B89, hence representing a separate genospecies. Compared to *B. chilensis* VA1, the type strain of the species, the four samples showed 94.10%–94.73% identity (Supporting Information 2: Figure S1).

**Figure 2:**
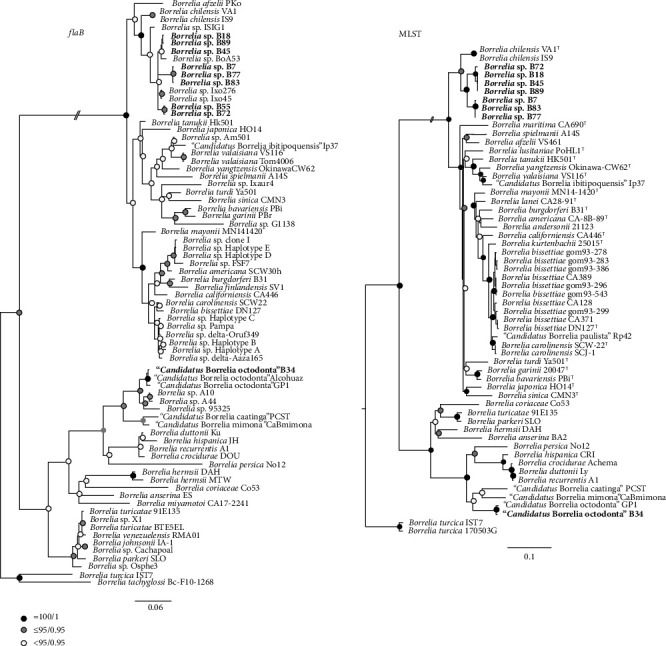
Consensus phylogenetic trees for *Borrelia flaB* gene and MLST analyses. Trees are drawn to scale with the scale bar indicating nucleotide substitutions per site. The positions of the detected haplotypes/strains are highlighted in bold.

The phylogenetic tree constructed for the *flaB* gene showed that the sequences obtained in this study clustered within the LDG and RFG groups. In fact, samples B7, B18, B45, B55, B72, B77, B83, and B89 formed a polytomy with LDG sequences previously retrieved from rodents (*Borrelia* sp. A53, GenBank: MN596014) or *Ixodes* (*Borrelia* sp. Ixo45, GenBank: MH187987; *Borrelia* sp. Ixo276, GenBank: MH178397) ticks in Chile, albeit with low statistical support. Moreover, this polytomy was closely related to a *Borrelia* sp. detected in *Ixodes* ticks from rodents in southern Argentina and to *B. chilensis*. In the RFG group, the sample B34 clustered into a well-supported monophyletic group with “*Ca*. B. octodonta.” With high statistical support, the MLST phylogeny showed that samples B7, B77, and B83 formed a monophyletic clade, sister to a clade consisting of samples B18, B45, B72, and B89. In turn, these samples were closely related to *B. chilensis*, also with strong statistical support. In the case of sample B34, the MLST phylogeny confirmed its close relatedness to “*Ca*. B. octodonta” (Figure 2).

## 4. Discussion

Tick-borne spirochetes are an emerging field of study in South America. For instance, two genospecies of the LDG, “*Candidatus* Borrelia paulista” and “*Candidatus* Borrelia ibitipoquensis,” and two of the RFG, “*Ca*. B. Caatinga” and “*Ca*. B. mimona,” have been described in Brazil [15–19]. Additionally, a species intermediate between the LDG and RFG, “*Candidatus* Borrelia mahuryensis,” was described from ticks in tropical French Guiana [5]. Here, two novel genospecies of the LDG were genetically identified in semiarid ecosystems of northern Chile, providing insights into the evolutionary history of *Borrelia* spirochetes in underexplored regions of the South American continent.

The transmission cycle of LDG *Borrelia* involves the infection of *Ixodes* ticks and their vertebrate hosts, which often include rodents. For example, in North America, phylogenetically closely related *Borrelia bissettiae*, *Borrelia californiensis*, and *Borrelia carolinensis* are transmitted by *Ixodes* spp. that parasitize primarily rodents [40, 41]. This ecological pattern seems to occur also in the prospected ecosystem, since the two novel genospecies of LDG *Borrelia* were both detected in *Ixodes* spp. associated with rodents and are phylogenetically related. Particularly, two species of *Ixodes* are known to infest rodents in the area where this study was conducted, namely *I*. *abrocomae* and *I*. *sigelos* [21, 42]. Therefore, it is reasonable to state that these species could be implicated in transmission cycles of the novel *Borrelia* genospecies. While the nymph of *I. abrocomae* remains undescribed, the female exhibits subtle morphological differences from *I. sigelos*, primarily in the anatomy of the capitulum and coxae [42]. In this study, species-level morphological identification of the analyzed tick specimens was not possible to achieve given that key diagnostic structures such as the capitulum or coxae were damaged. However, sequences of the mitochondrial 16S rRNA gene retrieved from *Borrelia*-positive ticks confirmed the species, indicating that they belonged either to *I*. *abrocomae* or *I*. *sigelos* (Supporting Information 1: Table S2).

After identifying *Borrelia*-positive ticks, the sequencing of *flaB* gene amplicons provided initial data to discern whether the spirochetes belonged to the LDG or RFG. Those sequences were also employed in a phylogeny that positioned the detected spirochetes into a polytomic group, possibly because of the shortness of the sequences. Despite the polytomy in the *flaB* tree, discrete groups conformed by the haplotypes characterized in this study and previously reported sequences from the region were clearly visible, including the two novel genospecies (Figure 2). The hypothesis of two novel genospecies of *B. burgdorferi* sensu lato, was further tested using genetic intraspecific thresholds after nucleotide pairwise comparisons of concatenated MLST gene alignments with strains of nine species of the LDG (Table 2), and through comparisons of genetic identities in the alignment constructed for the MLST phylogeny (Supporting Information 2: Figure S1). The fact that the intraspecific genetic divergence among fully characterized novel strains of our study exceeded the calculated threshold for other *Borrelia* spp., including *B. chilensis*, confirmed our hypothesis. For the novel strains with fewer sequenced genes, the MLST phylogenies validated their identity based on their close relatedness with strains for which the eight genes were sequenced. Indeed, the MLST phylogenetic trees defined two discrete clades, one composed of strains B18, B45, B72, and B89, and the other of strains B7, B77, and B83, which in turn clustered sister to *B. chilensis* (Figure 2). Noteworthy, the evolutionary relationship of these two novel genospecies with *B. chilensis* is interesting, since ticks positive for these *Borrelia* species branch into the same group as well. Indeed, *B. chilensis* was isolated from *I*. *stilesi*, a tick species that forms a monophyletic group with other *Ixodes* species endemic to the Southern Cone of South America, such as *I. abrocomae* and *I. sigelos* [21], which were collected in this study.

In addition to the LDG strains characterized in this study, we also detected DNA of RFG spirochete “*Ca*. B. octodonta,” which is transmitted by the soft tick *O. octodontus* [17]. The fact that DNA of “*Ca*. B. octodonta” was detected in an *Ixodes* and not in an *Ornithodoros* is not surprising, since the positive tick could have been feeding on a host harboring spirochetes in its bloodstream. If this reasoning is correct, it is notable that “*Ca*. B. octodonta” was present in *P. darwini* blood because it suggests that this rodent species would be susceptible to infection by this spirochete. However, *O. octodontus*, the known vector of “*Ca*. B. octodonta” has been found only on *Octodon degus* (Octodontidae) or within these rodent burrows [17, 22]. While the detection of the present study suggests that *“Ca. B. octodonta”* may have an ecology more complex than previously assumed, more evidence is needed to support this hypothesis.

## 5. Conclusions

This study contributes to adding two novel genospecies of the LDG and expands the knowledge on a previously detected genospecies of the RFG in Chile. While the detection and genetic characterization of *Borrelia* spirochetes in ticks provides valuable information regarding the circulation of genospecies, the study of LDG and RFG spirochetes in South America is still at an early stage. The isolation and in vitro cultivation of most genospecies that have been detected remain pending, limiting investigations into their biology. Filling this gap in the future will be crucial if we are to clarify the ecological roles that these spirochetes have and to endeavor into molecular diagnostics in animals and humans.

## Figures and Tables

**Table 1:** *Borrelia*-positive samples, sequenced genes (length), GenBank accession numbers, and *Borrelia* pubMLST database (https://pubmlst.org/organisms/borrelia-spp) assigned sequence type (ST), with allele numbers between brackets.

Sample id. (stage)	Locality	Collection date	Genes										
			Tick 16S rRNA (405 bp)	*flaB* (306 bp)	*clpA* (579 bp)	*clpX* (624 bp)	*nifS* (564 bp)	*pepX* (570 bp)	*pyrG* (603 bp)	*recG* (651 bp)	*rplB* (624 bp)	*uvrA* (570 bp)	Sequence type
LDG													
B7 (nymph)	FJNP	May 15, 2023	PV085741	PV082025	PV082026	PV082027	PV082028	PV082029	PV082030	PV082031	PV082032	—	—
B18 (nymph)	FJNP	May 19, 2023	PV085736	PV082048	PV082049	PV082050	PV082051	PV082052	PV082053	PV082054	PV082055	—	—
B45 (nymph)	FJNP	May 14, 2023	PV085739	PV082056	PV082057 [allele 340]	PV082058 [allele 300]	PV082059 [allele 265]	PV082060 [allele 296]	PV082061 [allele 307]	PV082062 [allele 335]	PV082063 [allele 290]	PV082064 [allele 301]	1198
B55 (nymph)	FJNP	Jan 17, 2023	—	PV082083	—	—	—	—	—	—	—	—	—
B72 (female)	FJNP	Apr 20, 2022	PV085737	PV082065	PV082066 [allele 341]	PV082067 [allele 301]	PV082068 [allele 266]	PV082069 [allele 297]	PV082070 [allele 309]	PV082071 [allele 336]	PV082072 [allele 291]	PV082073 [allele 302]	1199
B77 (female)	FJNP	Jan 15, 2022	PV085742	PV082033	PV082034 [allele 339]	PV082035 [allele 302]	PV082036 [allele 267]	PV082037 [allele 298]	PV082038 [allele 308]	PV082039 [allele 337]	PV082040 [allele 292]	PV082041 [allele 303]	1200
B83 (nymph)	FJNP	Jan 16, 2022	PV085743	PV082042	PV082043	PV082044	PV082045	PV082046	—	—	PV082047	—	—
B89 (female)	FJNP	May 16, 2023	PV085738	PV082074	PV082075	PV082076	PV082077	PV082078	PV082079	PV082080	PV082081	PV082082	—
RFG-													
B34 (nymph)	Tangue	Apr 30, 2023	PV085744	PV091804	—	PV091805 [allele 303]	—	PV091806 [allele 299]	—	PV091807 [allele 338]	PV091808 [allele 293]	—	—
B36 (nymph)	Tangue	Apr 30, 2023	PV085740	—	—	—	—	—	—	—	—	—	—

Abbreviations: bp, base pairs; FJNP, Fray Jorge National Park; LDG, lyme disease group; RFG, relapsing fever group.

**Table 2:** Genospecies and number of strains of *B. burgdorferi* sensu lato included to calculate intraspecific genetic identities of eight concatenated housekeeping genes downloaded from *Borrelia* PubMLST database.

Species	Min. %	No. of strains
*B. afzelii*	99.14	683
*B. bavariensis*	98.08	243
*B. bissettiae*	98.39	25
*B. burgdorferi*	98.22	1848
*B. carolinensis*	98.39	25
*B. garinii*	98.35	516
*B. lusitaniae*	98.58	51
*B. valaisiana*	99.31	112
*B. yangtzensis*	97.66	29

Note: Min. %, minimum percentage of genetic identity between strains of each species.

## Data Availability

The data that support the findings of this study are available from the corresponding author upon reasonable request.

## Supporting Information

Supporting Information 1 Table S1. Total number of Phyllotis darwini specimens (*n* = 57) from which ticks were collected and deposited in the “Colección Chilena de Garrapatas (CCG)” (larvae) or submitted to DNA extraction (nymphs and females). Abbreviation: FJNP, Fray Jorge National Park. Table S2. BLASTn results for tick mitochondrial 16S rRNA and flaB genes. Identical haplotypes are grouped for each gene.

Supporting Information 2 Figure S1. Heatmap matrix of pairwise sequence identities constructed for the MLST gene alignment of Borrelia spp. included in the phylogeny. Borrelia spp. detected in this study are highlighted in bold.
