# Supplementary material for: Multilocus Sequence Typing Unveils Two Novel Genospecies of Borrelia burgdorferi Sensu Lato in Ticks Infesting Cricetid Rodents of Northern Chile
Source: Transbound Emerg Dis. 2025 Jul 10;2025:8841276. doi: 10.1155/tbed/8841276 (PMC12271690; doi:10.1155/tbed/8841276)
Supplement: Supporting Information 1 — Table S1. Total number of Phyllotis darwini specimens (n = 57) from which ticks were collected and deposited in the “Colección Chilena de Garrapatas (CCG)” (larvae) or submitted to DNA extraction (nymphs and females). Abbreviation: FJNP, Fray Jorge National Park. Table S2. BLASTn results for tick mitochondrial 16S rRNA and flaB genes. Identical haplotypes are grouped for each gene. [file 8841276.f1.docx]

**SUPPLEMENTARY TABLES**

Table S1. Total number of *Phyllotis darwini* specimens (n=57) from which ticks were collected for deposit in the "Colección Chilena de Garrapatas (CCG)" (larvae) and DNA extraction (nymphs and females). Abbreviation: PNJ, Fray Jorge National Park.

| **Specimen** | **Larvae** | **Nymphs** | **Females** | **Extraction  DNA codes** | **Borrelia detection** | **Locality** | **Date** |
| --- | --- | --- | --- | --- | --- | --- | --- |
| 1 | 0 | 1 | 0 | B1 | No | FJNP | 14-05-2023 |
| 2 | 1 | 1 | 0 | B2 | No | FJNP | 14-05-2023 |
|  |  | 1 | 0 | B3 | No | FJNP | 14-05-2023 |
|  |  | 1 | 0 | B4 | No | FJNP | 14-05-2023 |
| 3 | 0 | 1 | 0 | B5 | No | FJNP | 14-05-2023 |
| 4 | 2 | 1 | 0 | B6 | No | FJNP | 15-05-2023 |
| 5 | 0 | 1 | 0 | B7 | *Borrelia* sp. B7 | FJNP | 15-05-2023 |
|  |  | 1 | 0 | B8 | No | FJNP | 15-05-2023 |
|  |  | 1 | 0 | B9 | No | FJNP | 15-05-2023 |
|  |  | 1 | 0 | B10 | No | FJNP | 15-05-2023 |
|  |  | 1 | 0 | B11 | No | FJNP | 15-05-2023 |
| 6 | 0 | 1 | 0 | B12 | No | FJNP | 16-05-2023 |
| 7 | 0 | 1 | 0 | B13 | No | FJNP | 18-05-2023 |
|  |  | 1 | 0 | B14 | No | FJNP | 18-05-2023 |
|  |  | 1 | 0 | B15 | No | FJNP | 18-05-2023 |
|  |  | 1 | 0 | B16 | No | FJNP | 18-05-2023 |
|  |  | 1 | 0 | B17 | No | FJNP | 18-05-2023 |
| 8 | 0 | 1 | 0 | B18 | *Borrelia* sp. B18 | FJNP | 19-05-2023 |
| 9 | 0 | 1 | 0 | B19 | No | FJNP | 25-05-2023 |
| 10 | 1 | 1 | 0 | B20 | No | FJNP | 27-05-2023 |
| 11 | 0 | 1 | 0 | B21 | No | Tangue | 30-04-2023 |
| 12 | 0 | 1 | 0 | B22 | No | FJNP | 19-01-2023 |
| 13 | 4 | 1 | 0 | B23 | No | FJNP | 19-01-2023 |
| 14 | 0 | 1 | 0 | B24 | No | FJNP | 19-01-2023 |
| 15 | 10 | 1 | 0 | B25 | No | FJNP | 20-01-2023 |
|  |  | 1 | 0 | B26 | No | FJNP | 20-01-2023 |
| 16 | 3 | 1 | 0 | B27 | No | FJNP | 20-01-2023 |
| 17 | 2 | 1 | 0 | B28 | No | FJNP | 20-01-2023 |
| 18 | 1 | 1 | 0 | B29 | No | FJNP | 20-01-2023 |
| 19 | 4 | 1 | 0 | B30 | No | FJNP | 22-01-2023 |
| 20 | 1 | 1 | 0 | B31 | No | FJNP | 22-01-2023 |
| 21 | 0 | 1 | 0 | B32 | No | FJNP | 22-01-2023 |
| 22 | 0 | 1 | 0 | B33 | No | Tangue | 30-04-2023 |
|  |  | 1 | 0 | B34 | *Borrelia* sp. B34 | Tangue | 30-04-2023 |
|  |  | 1 | 0 | B35 | No | Tangue | 30-04-2023 |
|  |  | 1 | 0 | B36 | *Borrelia* sp. B36 | Tangue | 30-04-2023 |
|  |  | 1 | 0 | B37 | No | Tangue | 30-04-2023 |
|  |  | 1 | 0 | B38 | No | Tangue | 30-04-2023 |
|  |  | 1 | 0 | B39 | No | Tangue | 30-04-2023 |
|  |  | 1 | 0 | B40 | No | Tangue | 30-04-2023 |
|  |  | 1 | 0 | B41 | No | Tangue | 30-04-2023 |
| 23 | 0 | 1 | 0 | B42 | No | Tangue | 30-04-2023 |
| 24 | 0 | 1 | 0 | B43 | No | Tangue | 01-05-2023 |
| 25 | 0 | 1 | 0 | B44 | No | Tangue | 02-05-2023 |
| 26 | 0 | 1 | 0 | B45 | *Borrelia s*p. B45 | FJNP | 14-05-2023 |
| 27 | 0 | 1 | 0 | B46 | No | FJNP | 22-01-2023 |
| 28 | 1 | 1 | 0 | B47 | No | FJNP | 16-05-2023 |
| 29 | 0 | 1 | 0 | B48 | No | Tangue | 01-05-2023 |
|  |  | 1 | 0 | B49 | No | Tangue | 01-05-2023 |
|  |  | 1 | 0 | B50 | No | Tangue | 01-05-2023 |
|  |  | 1 | 0 | B51 | No | Tangue | 01-05-2023 |
|  |  | 1 | 0 | B52 | No | Tangue | 01-05-2023 |
| 30 | 0 | 1 | 0 | B53 | No | FJNP | 17-05-2023 |
|  |  | 1 | 0 | B54 | No | FJNP | 17-05-2023 |
| 31 | 1 | 1 | 0 | B55 | Only *flaB* | FJNP | 17-01-2023 |
|  |  | 1 | 0 | B56 | No | FJNP | 17-01-2023 |
| 32 | 1 | 0 | 1 | B57 | No | FJNP | 18-05-2023 |
| 33 | 1 | 1 | 0 | B58 | No | FJNP | 21-05-2023 |
| 34 | 0 | 1 | 0 | B59 | No | FJNP | 18-05-2023 |
| 35 | 0 | 0 | 1 | B60 | No | FJNP | 19-05-2023 |
| 36 | 0 | 0 | 1 | B61 | No | FJNP | 19-05-2023 |
| 37 | 1 | 1 | 0 | B62 | No | FJNP | 21-05-2023 |
| 38 | 0 | 1 | 0 | B63 | No | Tangue | 02-05-2023 |
| 39 | 0 | 0 | 1 | B64 | No | FJNP | 16-05-2023 |
| 40 | 0 | 0 | 1 | B65 | No | FJNP | 15-10-2021 |
|  |  | 0 | 1 | B66 | No | FJNP | 15-10-2021 |
| 41 | 0 | 1 | 0 | B67 | No | FJNP | 15-10-2021 |
|  |  | 0 | 1 | B68 | No | FJNP | 15-10-2021 |
| 42 | 0 | 0 | 1 | B69 | No | FJNP | 16-10-2021 |
| 43 | 0 | 0 | 1 | B70 | No | FJNP | 23-01-2022 |
|  |  | 0 | 1 | B71 | No | FJNP | 23-01-2022 |
| 44 | 0 | 0 | 1 | B72 | *Borrelia* sp. B71 | FJNP | 20-04-2022 |
| 45 | 1 | 0 | 1 | B73 | No | FJNP | 15-04-2022 |
| 46 | 1 | 0 | 1 | B74 | No | FJNP | 15-04-2022 |
| 47 | 1 | 1 | 0 | B75 | No | FJNP | 19-04-2022 |
|  |  | 1 | 0 | B76 | No | FJNP | 19-04-2022 |
| 48 | 0 | 0 | 1 | B77 | *Borrelia* sp. B77 | FJNP | 15-01-2022 |
| 49 | 0 | 1 | 0 | B78 | No | FJNP | 15-01-2022 |
|  |  | 1 | 0 | B79 | No | FJNP | 15-01-2022 |
| 50 | 2 | 0 | 1 | B80 | No | FJNP | 17-01-2022 |
| 51 | 0 | 1 | 0 | B81 | No | FJNP | 17-01-2022 |
| 52 | 1 | 1 | 0 | B82 | No | FJNP | 15-01-2022 |
| 53 | 2 | 1 | 0 | B83 | *Borrelia* sp. B83 | FJNP | 16-01-2022 |
| 54 | 1 | 1 | 0 | B84 | No | FJNP | 16-07-2022 |
|  |  | 1 | 0 | B85 | No | FJNP | 16-07-2022 |
| 55 | 0 | 1 | 0 | B86 | No | FJNP | 22-07-2022 |
| 56 | 0 | 1 | 0 | B87 | No | FJNP | 18-07-2022 |
| 57 | 1 | 0 | 1 | B88 | No | FJNP | 16-05-2023 |
|  |  | 0 | 1 | B89 | *Borrelia* sp. B89 | FJNP | 16-05-2023 |
|  |  | 0 | 1 | B90 | No | FJNP | 16-05-2023 |

Table S2.  BLASTn results of tick mitochondrial 16S rRNA and *flaB* genes.

| **Samples codes** | **BLASTn results for tick mitochondrial 16S rRNA** |
| --- | --- |
| **Haplotype 1**  B18 (nymph)  B72 (female)  B89 (female) | 99.26% (403/406), 100% query cover, 0 E-value, 0 gaps, similar with *Ixodes abrocomae* (GU188043) collected on *Abrothrix olivacea* in Coquimbo region, Chile. |
| **Haplotype 2**  B45 (nymph) | 99.75% (404/405), 100% query cover, 0 E-value, 0 gaps, similar with *Ixodes abrocomae* (GU188043) collected on *Abrothrix olivacea* in Coquimbo region, Chile. |
| **Haplotype 3**  B7 (nymph)  B36 (nymph)  B77 (female)  B83 (nymph) | 100% (406/406), 100% query cover, 0 E-value, 0 gaps, similar with *Ixodes* cf. *sigelos* (MH183256) collected on *Phyllotis darwini* in Bosque de Fray Jorge National Park, Chile. |
| **Haplotype 4**  B34 (nymph) | 99.75% (405/406), % query cover, 0 E-value, 0 gaps, similar with *Ixodes* cf *sigelos* (MH183256) collected on *Phyllotis darwini* in Bosque de Fray Jorge National Park, Chile. |
| **Samples codes** | **BLASTn results for *flaB* gene** |
| Lyme group | |
| **Haplotype 1**  B7, B77, B83 | 98.04% (300/306), 100% query cover, 0 E-value, 0 gaps, similar to Uncultured *Borrelia* sp. clone BoA53 (MN596014) characterized from blood of *Oligoryzomys longicaudatus*  in Bosque de Fray Jorge National Park, Chile. |
| **Haplotype 2**  B18, B45, B89 | 99.35% (304/306), 100% query cover, 0 E-value, 0 gaps, similar to *Borrelia* sp. isolate Ixo276 (MH178397) characterized from *Ixodes* sp. sigelos group in Mocha Island, Chile. |
| Haplotype 3  B55, B72 | 99.02% (303/306), 100% query cover, 0 E-value, 0 gaps, similar to *Borrelia* sp. isolate Ixo276 (MH178397) characterized from *Ixodes* sp. sigelos group in Mocha Island, Chile. |
| Relapsing fever group | |
| B34 | 100% (300/300), 100% query cover, 0 E-value, 0 gaps, identical with Uncultured *Borrelia* sp. clone Alcohuaz (MW981443) characterized from *Ornithodoros octodontus* in Alcohuaz, Chile. |
